# ResNeXt-Based Rescoring Model for Proteoform Characterization in Top-Down Mass Spectra

**DOI:** 10.1007/s12539-025-00701-x

**Published:** 2025-05-17

**Authors:** Jiancheng Zhong, Yicheng Luo, Chen Yang, Maoqi Yuan, Shaokai Wang

**Affiliations:** 1 College of Information Science and Engineering, Hunan Normal University, Changsha, 410081 China; 2 Department of Mathematics, Hong Kong University of Science and Technology, 999077 Hong Kong SAR, China

**Keywords:** Proteoform characterization, Rescoring, Deep learning

## Abstract

**Abstract:**

In top-down proteomics, the accurate identification and characterization of proteoform through mass spectrometry represents a critical objective. As a result, achieving accuracy in identification results is essential. Multiple primary structure alterations in proteins generate a diverse range of proteoforms, resulting in an exponential increase in potential proteoform. Moreover, the absence of a definitive reference set complicates the standardization of results. Therefore, enhancing the accuracy of proteoform characterization continues to be a significant challenge. We introduced a ResNeXt-based deep learning model, PrSMBooster, for rescoring proteoform spectrum matches (PrSM) during proteoform characterization. As an ensemble method, PrSMBooster integrates four machine learning models, logistic regression, XGBoost, decision tree, and support vector machine, as weak learners to obtain PrSM features. The basic and latent features of PrSM are subsequently input into the ResNeXt model for final rescoring. To verify the effect and accuracy of the PrSMBooster model in rescoring proteoform characterization, it was compared with the characterization algorithm TopPIC across 47 independent mass spectrometry datasets from various species. The experimental results indicate that in most mass spectrometry datasets, the number of PrSMs obtained after rescoring with PrSMBooster increases at a false discovery rate (FDR) of 1%. Further analysis of the experimental results confirmed that PrSMBooster improves the accuracy of PrSM scoring, generates more mass spectrometry characterization results, and demonstrates strong generalization ability.

**Graphical Abstract:**

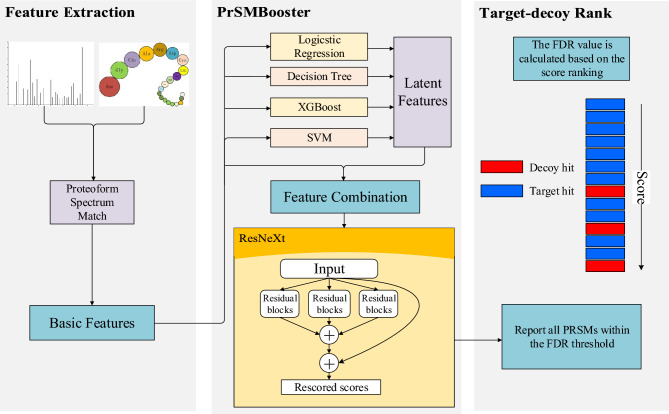

## Introduction

Proteoform characterization is essential for revealing the functional diversity of proteins and their involvement in biological processes [1–3]. Proteins with multiple primary structure alterations (PSA) produce a wide array of proteoforms, leading to a combinatorial expansion in possible proteoforms. As a result, accurately aligning tandem mass spectra with candidate proteoforms represents a considerable challenge in this domain.

At present, numerous tools have been developed for top-down proteoform characterization. These methods generally fall into two categories, expanding proteoform database method and blind search method for PSA. The first method involves searching expanded protein sequence databases, integrating theoretical protein sequences with preset modification information for proteoform characterization. The blind PSA search method avoids generating every possible proteoform sequence. Instead, it directly compares the experimental mass spectrum (MS) with a reference protein sequence. Together, these approaches have greatly advanced top-down proteoform characterization. Zamdborg et al. [4] introduced the ProSight PTM. The team used the shotgun annotation database strategy to construct proteoform database that includes PSA. ProSight PTM identified intact proteins through searches conducted within this proteoform database. The BIG Mascot search engine, developed by Karabacak et al. [5], improves fragmentation and data processing methods. Théberge et al. [6] utilized an LTQ-Orbitrap mass spectrometer and the BUPID-top-down algorithm to characterize proteoforms. For unidentified proteins, the BUPID-top-down algorithm searches the database for the most similar protein sequences, accelerating the process using a heuristic model. Li et al. [7] proposed the ProteinGoggle method. When customizing the database, ProteinGoggle incorporates the selected post-translational modifications (PTMs) and amino acid variations into the proteoform database to identify proteoforms. MetaMorpheus [8] is an open-source proteomics database search software developed by Solntsev et al., which adds the capability of discovering global PTMs, thereby enhancing the identification performance of proteoforms. Toby et al. [9] created TDPortal, which uses the Fourier transform MS data to identify proteins.

In contrast, blind PSA search methods directly match experimental MS to theoretical MS derived from reference protein sequences. For example, Tsai et al. [10] developed PIITA for top-down tandem MS de novo peptide sequencing. This algorithm is independent of precursor ions and first preprocesses all raw files in FASTA format using the InSilico-Spectro library, generating a new file containing only fragment ion masses to shorten search time. Subsequently, the matching score for fragment ions is calculated for each tandem mass spectrum. Frank et al. [11] introduced a dynamic programming algorithm to optimize the alignment path between MS data and protein sequences. This spectral alignment method enables the characterization of proteoforms with multiple modifications. The MS-Align algorithm, developed by Liu et al. [12], is capable of detecting unknown PTMs. They also introduced a method to evaluate the statistical significance of proteoform spectrum matches (PrSMs). Subsequently, Cai et al. [13] proposed MASH Suite Pro, an integrated software with a graphical user interface, offering functions such as protein identification, characterization, quantification, visual verification, and support for interface customization. This software is designed for top-down proteomics data analysis. The algorithm was further improved by the team through the development of MS-Align-E [14], which can identify proteins with multiple PTMs. It employs a dynamic programming algorithm to identify the longest path in the mass spectrum grid based on the number of PTMs and is utilized to identify both unknown and known modification types in proteins. The team subsequently developed TopPIC [15], an integrated tool combining algorithms for candidate protein filtering, spectral alignment, *e*-value computation, and Bayesian modeling to characterize unknown PSAs. TopPIC uses deconvoluted top-down MS to effectively identify and characterize complex proteoforms [16].

Machine learning methods have also been utilized in this field, exemplified by pTop, developed by Sun et al. [17, 18], which employs a model that integrates multiple MS features. This method uses a support vector machine for online training, improving the accuracy of precursor ion detection to facilitate proteoform identification.

**Fig. 1:**
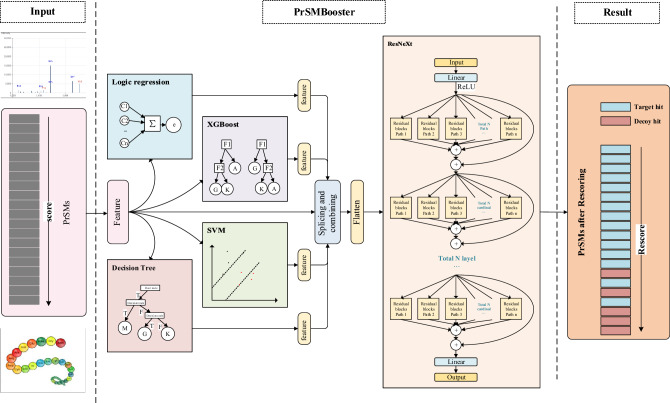
The Pipeline of PrSMBooster

Graph-based algorithms have been proposed by researchers, such as the approach by Vyatkina et al. [19], which proposed T-Bruijn graphs, an adaptation of the commonly used A-Bruijn graphs from genomics. In a following study, T-Bruijn graphs were employed to calculate and assemble de novo amino acid sequences generated through the Twister approach [20]. Park et al. [21] proposed Informed-Proteomics, which utilizes a sequence graph method to investigate potential proteoforms. Kou et al. [22] introduced TopMG, a mass graph-based tool for proteoform characterization that formulates this task as a mass graph alignment problem, solved through the dynamic programming method.

Proteoform characterization methods are primarily based on built-in scoring algorithms, such as Bayesian-based MIScore, spectral probability approaches, and dynamic programming algorithms. However, when dealing with highly heterogeneous or complex MS, the protein sequences reported in PrSM are often characterized by multiple PSA, including terminal truncation, variable PTMs, and unknown mass shifts. PrSM scoring methods, such as spectral probability, primarily generate confidence scores and rankings based on the number of matching peaks, which does not reliably reflect the accuracy of all PrSMs. Several important PrSMs are sometimes overlooked in the target-decoy strategy as a result of their low rankings. To overcome this limitation, the PrSMBooster model was introduced, integrating four traditional machine learning models as weak learners to extract the latent features of PrSM, and ultimately rescoring PrSM with the deep learning model ResNext. The rescoring process is employed as a post-processing procedure in proteoform characterization. Post-processing procedure refers to a series of strategies that adjust results following the application of a method for data processing, remaining independent of the initial characterization process.

PrSMBooster seeks to improve characterization by accurately rescoring PrSMs supported by substantial MS-based evidence, thus enhancing their rankings within the target-decoy search strategy. We conducted a comparative analysis across 47 cross-species MS datasets to assess the rescoring results of PrSMBooster in comparison with TopPIC. PrSMBooster further validates the effectiveness of the model in enhancing characterization accuracy through the reporting of a greater amount of PrSMs.

**Table 1:** Summary of the MS datasets

PRIDE ID	Species	Description	Abbreviations	Subdataset	
-	Zebrafish	Zebrafish	FB_TO	FB_TO_1	FB_TO_2
				FB_TO_3	
PXD026178 [23]	Human	Data for the Blood Proteoform Atlas:DS-75: Platelet:Purchased from IR	Human_L	Human_L1	Human_L2
				Human_L3	Human_L4
PXD026128 [23]	Human	Data for the Blood Proteoform Atlas:DS-06: Neutrophil:Peripheral Blood	Human_0	Human_01	Human_02
				Human_03	Human_04
PXD029703 [24]	Human	Human colorectal cancer cell	Human_E	Human_E1	Human_E2
				Human_E3	Human_E4
				Human_E5	Human_E6
PXD042298 [25]	Mouse	Mouse brain integral membrane	AH	AH_1	AH_2
				AH_3	AH_4
				AH_5	AH_6
PXD018772 [26]	Tenebrio molitor	Tenebrio molitor	TM	TM_1	TM_2
				TM_3	TM_4
				TM_5	
PXD017382 [27]	Pisum sativum	Pisum sativum	PS	PS_1	PS_2
				PS_3	PS_4
				PS_5	PS_6
				PS_7	
PXD034368 [28]	Arabidopsis thaliana	Arabidopsis thaliana (mouse-ear cress)	AT	AT_1	AT_2
				AT_3	AT_4
				AT_5	AT_6
PXD046651 [29]	Yeast	Saccharomyces cerevisiae and Candida albicans (yeast)	Yeast	Yeast_1	Yeast_2
				Yeast_3	Yeast_4
				Yeast_5	Yeast_6

## Method

The primary goal of proteoform characterization algorithms based on top-down MS is the identification of PrSM. PrSMBooster is constructed by integrating a deep learning framework. During the post-processing stage of proteoform characterization, PrSM is rescored by the model. PrSMBooster improves the ranking of more credible target matches, while decoy matches are ranked lower due to reduced scores after rescoring. Figure 1 presents a schematic diagram of the PrSMBooster pipeline, which primarily includes a feature extraction module and a ResNeXt-based rescoring module.

### Basic Feature Extraction

PrSMBooster acquires basic features by entering intermediate results from TopPIC characterization. In the intermediate results, it does not provide any annotation information regarding whether PrSM is a target match or a decoy match. Based on this, PrSMBooster analyzes PrSM and extracts the following basic features.

1. Number of matching peaks. The number of matching peaks in PrSM refers to the matches found between peaks in the experimental MS and theoretical MS. This feature is used to assess consistency between experimental data and theoretical data by comparing the experimentally measured MS with the theoretical MS of a known protein. The experimentally obtained MS represents the signal measured after ionizing the sample using a mass spectrometer. The theoretical MS is generated by calculating the amino acid sequence, predicting the ion fragment mass-to-charge ratios, and subsequently constructing the theoretical MS. A higher number of matching peaks indicates greater consistency between experimental and theoretical data, facilitating protein sequence and structure determination. In summary, a higher number of matching peaks generally corresponds to increased accuracy in proteoform identification and characterization.
2. Number of matching fragments. The number of matching fragments refers to the count of fragment ions in the experimental MS that correspond to those in the theoretical MS, serving as a feature for assessing proteoform characterization accuracy. MS experiments typically yield ion fragments that reflect both the amino acid sequence and any possible modifications of the protein. These experimental fragments are compared with theoretically predicted fragments, derived by calculating the amino acid sequence of known proteins and predicting the resultant fragment ions in the MS. A larger number of matching fragments typically indicates greater alignment between experimental data and theoretical predictions, thereby enhancing the reliability of proteoform characterization.
3. Number of PTMs. PTMs alter protein structure, function, and activity. These modifications include phosphorylation, methylation and others. The study of PTMs is crucial for understanding protein functionality and regulating cellular processes. In MS analysis, detected modification patterns and positional information contribute to insights into protein function and regulatory mechanisms. The presence of multiple modifications in a protein can alter the position and intensity of the peaks in MS. Therefore, understanding the number of PTMs facilitates more accurate interpretation of MS.
4. Original rank indicator. For proteoform characterization algorithms that employ the target-decoy strategy to control FDR, a ranking is required, representing the certainty and reliability of the characterization algorithm for the reported PrSMs. The characterization algorithm TopPIC applies spectral probability calculations to determine the expected error value (*e*-value) for all PrSMs. Based on the *e*-value, PrSM results are sorted in ascending order, and then FDR of each PrSM under the target-decoy strategy is calculated. Thus, a smaller *e*-value of PrSM indicates greater credibility. TopPIC additionally offers *p*-value, an indicator of PrSM unreliability. Although *p*-value is positively correlated with *e*-value, it remains within a probability range of 0 to 1, unlike the potentially high e-value, which can reach up to $1 \times 10^{300}$. Thus, when *p*-value of a PrSM equals 1, it indicates that the identification algorithm deems this PrSM unreliable. This study incorporates both *e*-value and *p*-value features into PrSMBooster model, where *e*-value is log-transformed. Through logarithmic transformation, the e-value indicator is mapped to a normalized range.

**Fig. 2:**
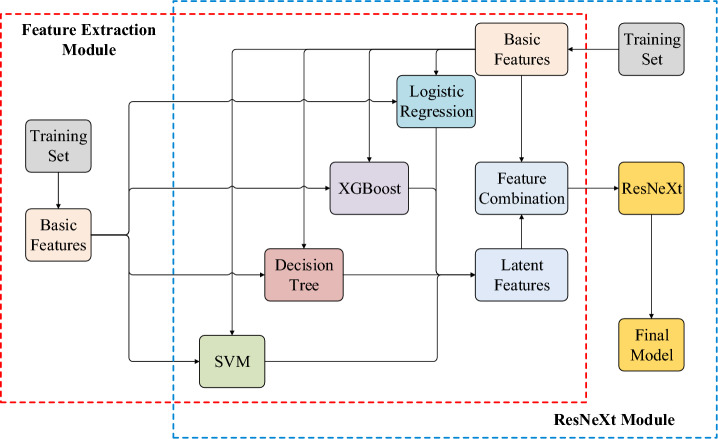
The model training of PrSMBooster

### Rescoring Model

As an ensemble learning model, PrSMBooster integrates four traditional machine learning algorithms. It extracts latent features from the basic PrSM features. The basic and latent features are ultimately predicted by ResNeXt, resulting in the calculation of PrSM score.

By integrating these models for extracting the latent features, PrSMBooster leverages the strengths of each: logistic regression for linearity, XGBoost for complex nonlinearity, decision tree for boundary clarity, and SVM for high-dimensional feature mapping. This ensemble approach not only reduces the risk of overfitting, which is common in individual models, but also ensures better generalization across diverse datasets. Additionally, the integration of multiple models improves the model’s capacity to capture both linear and high-dimensional nonlinear relationships of features, thus enhancing PrSMBooster’s robustness and adaptability.

#### Latent Feature Extraction Module

The experimental inputs consist of basic features including the matching peaks, matching fragments, number of PTMs, *e*-value and *p*-value. The features extracted from PrSM are converted into one-dimensional vectors and input into traditional non-deep learning models. The following section will present these machine learning models with regard to their characteristics and parameters.

1. Logistic regression. The model predicts the probability score of PrSM matching the target by processing the linear relationship between the basic features and the PrSM labels. The parameter configuration is as follows. The "fit_intercept" parameter allows the model to add constants to the decision function. Since basic features of PrSM are used directly, we do not regularize the data features, and "normalize" is set to "False" accordingly. "N_jobs" is set to "None", limiting computation to a single CPU core, and "positive" is set to "False", meaning no constraint is applied to keep regression coefficients positive. In the experiment, basic features are predicted to yield a single probability value, which is then integrated into the PrSM features.
2. XGBoost. This machine learning model generates prediction from PrSM features, using the predicted value as a latent PrSM feature. To enable the XGBoost model to achieve an ideal fitting, it is essential to set appropriate learning rates and the depth of decision trees. In the experiment, properly setting the maximum depth of the decision tree helps prevent overfitting. In this model, the "max_depth" parameter is 5. The "learning_rate", representing the shrinkage during decision tree generation, is set to 0.1. The parameter "n_estimators" represents the number of weak estimators, set to 160 in this model.
3. Decision tree. This model is effective at handling nonlinear relationships and can process unnormalized PrSM data directly. In this experiment, the decision tree model is configured with a maximum depth of 4 layers, and the Gini coefficient is employed to select the split nodes. Other parameters of the model were left at scikit-learn’s default settings to provide a standard implementation without extra tuning.
4. SVM. SVM remains widely used in machine learning and, as a classic supervised model, is well-suited for classification tasks. Select the polynomial kernel "poly" as the kernel function. The kernel function parameter "gamma" is 1. The parameter "*c*" is used to control the penalty coefficient of the loss function, which is 0.1 here. To enable probability estimation, set the parameter "probability" to "True" and configure "max_iter" to 10,000 to define the maximum number of solver iterations. The robustness of SVM makes it especially useful for extracting latent features.

#### Final Rescoring Module Based on ResNeXt

ResNeXt incorporates group convolution into the residual network [30, 31]. PrSMBooster employs ResNeXt to perform the final comprehensive rescoring. The residual connections in ResNeXt provide a simple and effective means of increasing network depth. Deeper networks can capture more complex nonlinear relationships, thereby offering accurate scoring functions for proteoform characterization. ResNeXt’s group operations provide an advantage over standard ResNet models by reducing computational complexity and accelerating training speed with the same condition of parameters.

Residual learning can be extended across multiple layers. The structure of a residual block in ResNet is defined as follows.

1 $$\begin{aligned} y=F(x, W+x) \end{aligned}$$

Here, *x* denotes the model input, which includes all features of PrSM, while *y* represents the predicted output of the model. The function *F* corresponds to the residual map. This study employs the ResNeXt model composed of three layers of residual blocks. The model configuration specifies 64 neurons per layer, with ReLU applied as the activation function in both the input layers and residual blocks of ResNeXt.

#### PrSMBooster Model Training

The PrSMBooster training process consists of two modules, as illustrated by the model structure in Fig. 2. The first module is the feature extraction module, where the training set is used to train traditional machine learning models separately for feature extraction. In this module, each PrSM data point generates a 4-dimensional basic feature vector. During training, PrSMs are labeled as either targets or decoys, but these labels are not available during the subsequent rescoring prediction process. The basic feature vectors are then input into logistic regression, XGBoost, decision tree, and SVM models to extract latent features. These latent features are obtained from the weak learner’s prediction scores, expanding the original 4-dimensional feature vector into an 8-dimensional vector, which includes both the 4 basic features and the 4 latent features.

The second module is the ResNeXt module, where the extracted features are integrated. The 8-dimensional feature vectors are fed into the ResNeXt model for further training. ResNeXt allows for parallel processing of input features through multiple residual blocks. The cardinality of the ResNeXt model refers to the number of parallel convolution paths in each residual block, which processes the input features independently. In PrSMBooster, the cardinality is set to 8, with each group containing 9 residual blocks. The model is optimized using the Adam optimizer, with a batch size of 100. Compared to standard stochastic gradient descent, Adam offers advantages such as adaptive learning rates, momentum estimation, and more flexible hyperparameter tuning. The learning rate is initially set to 0.001, and the model is trained for 50 epochs.

**Fig. 3:**
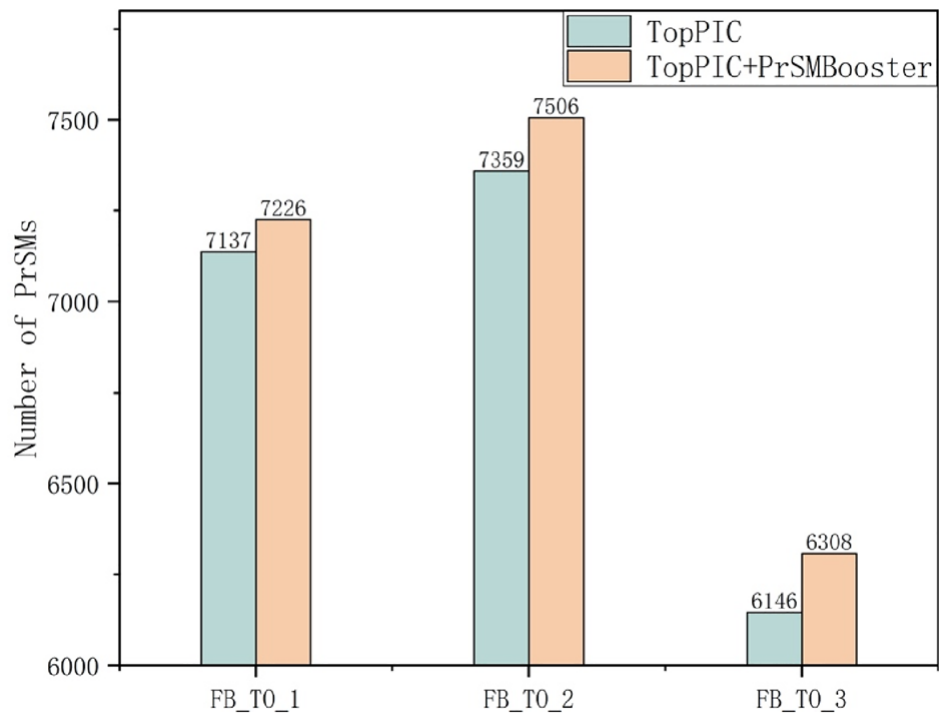
The PrSM results for FB_TO dataset

**Fig. 4:**
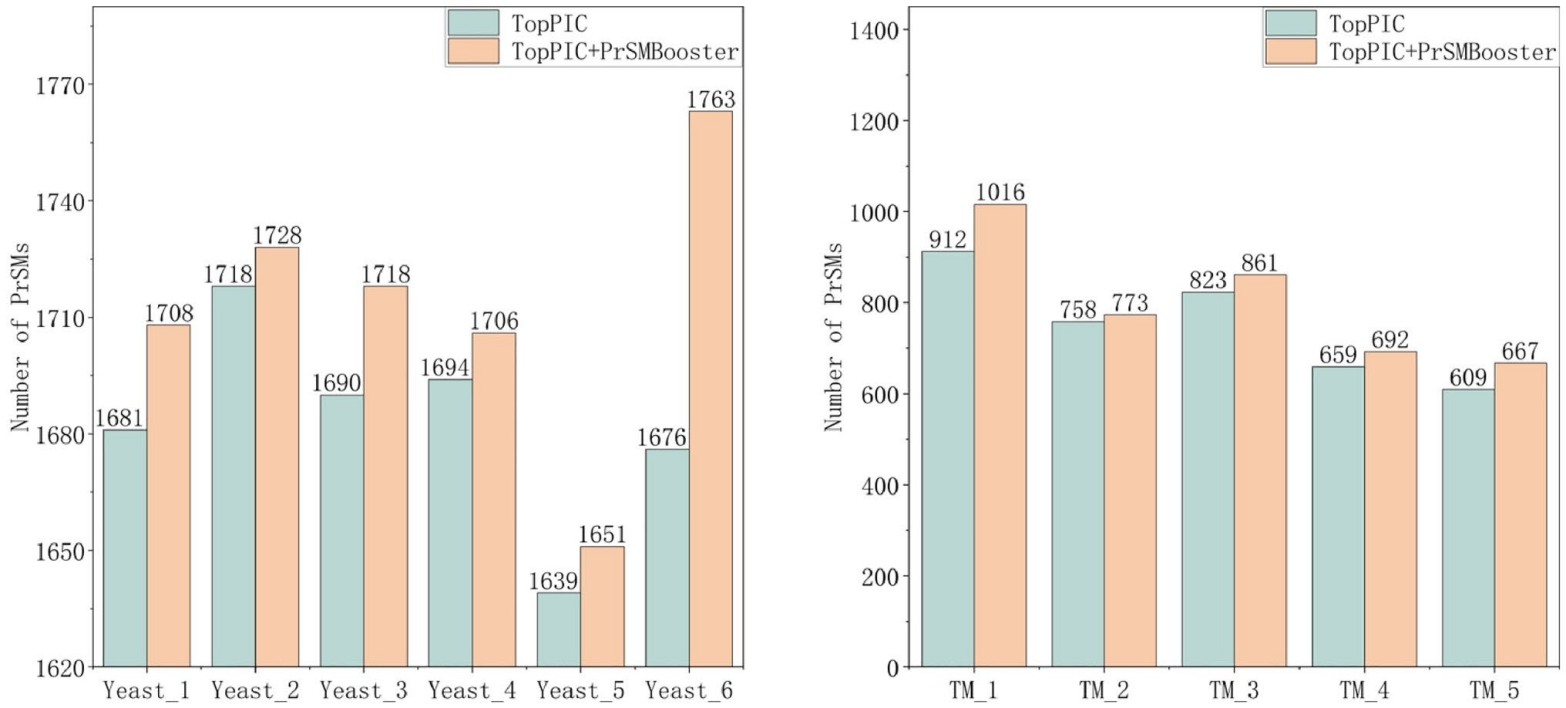
The PrSM results for Yeast (left) and TM (right) datasets

## Results and Discussion

### Dataset

The experiment was conducted using Python 3.6, the scikit-learn machine learning library, and the PyTorch deep learning framework.

The MS datasets used in this study were obtained from the European Bioinformatics Institute and include raw MS files from different species, such as yeast, pisum sativum, mouse, tenebrio molitor, arabidopsis thaliana and human. Additionally, the zebrafish dataset was sourced from the published literature [32]. A summary of the datasets is presented in Table 1. The top-down MS identification tool, TopPIC (v1.6.2), was used to characterize proteoforms in various MS. The TopPIC results reveal that the number of PrSMs identified in these MS datasets ranges from 9 to 9,019. PrSMBooster was trained on 12 MS datasets from human and zebrafish, with the remaining 47 datasets serving as test sets for prediction. Additionally, MSconvert, TopFD (v1.6.2), and TopPIC were employed for MS format conversion, deconvolution, and identification.

### Evaluation Criteria

PrSMBooster rescores and sorts the PrSM results based on the target-decoy strategy. First, the results are sorted by score, and then the FDR is calculated for each PrSM. This study employed a commonly used method for calculating the FDR. The formula used to calculate FDR for PrSM is as follows.

2 $$\begin{aligned} r_{\textrm{FD},i}=\frac{n_{\textrm{decoy},i}}{n_{\textrm{target},i}} \end{aligned}$$

Here, $n_{\textrm{decoy},i}$ denotes the quantity of PrSMs that match the decoy with the score ranks before *i*, while $n_{\textrm{target},i}$ denotes the quantity of PrSMs that match the target with the score ranks before *i*.

Finally, targets matching PrSMs with score below the set threshold or FDR below 0.01 are filtered, and the final results are reported based on the PrSMBooster rescoring score.

### Analysis of Impact of Rescoring

As a post-processing step in proteoform characterization, the rescoring method yields satisfactory results across 47 datasets from multiple species. PrSMBooster can effectively rescore input data from various species, demonstrating the model’s generalization ability. According to the number of candidate PrSMs in the TopPIC characterization process, the dataset is divided into three categories with 100 and 5000 as the boundaries.

#### Comparison of the Number of PrSMs

Figure 3 demonstrates an increase in outcomes following the application of PrSMBooster rescoring, particularly evident in FB_TO datasets. For three large datasets with more than 5000 candidate PrSMs, the number of reported PrSMs increases by 89, 147, and 162, respectively, after applying PrSMBooster.

Datasets with PrSM numbers between 100 and 5000 can also be improved after rescoring by PrSMBooster. Figure 4 presents the datasets for Yeast and TM. In Yeast dataset, larger improvements were observed across most subsets, with the exception of Yeast_2 and Yeast_5. Among them, Yeast_2 showed the smallest increase, with 10 additional PrSMs, whereas Yeast_6 exhibited the largest increase, adding 87 PrSMs.

In the datasets with PrSM numbers between 100 and 5000, most results showed improvements of over 8%. The majority of datasets reported PrSM counts of 30 or more. Notably, dataset TM_1 exhibited the largest increase, adding 104 PrSMs.

**Fig. 5:**
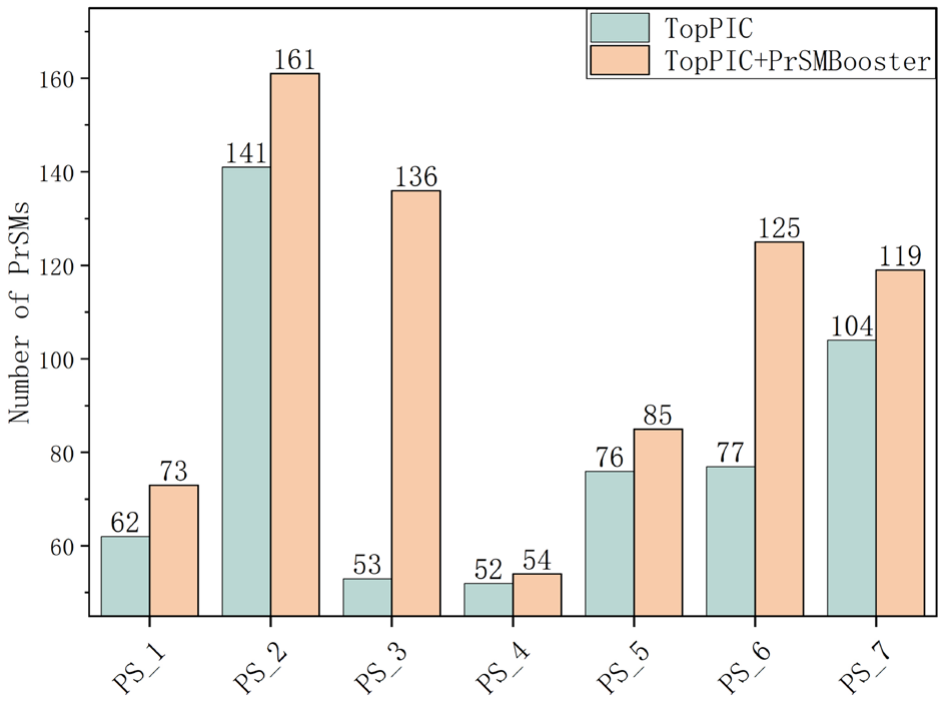
The PrSM results for PS dataset

Figure 5 presents the PS dataset, revealing a distinct trend, while large and medium-sized datasets showed advantages in the number of increase, datasets with less than 100 PrSMs exhibited more improvement ratios. With the exception of dataset PS_4, all datasets achieved improvement rates of up to 10%.

**Fig. 6:**
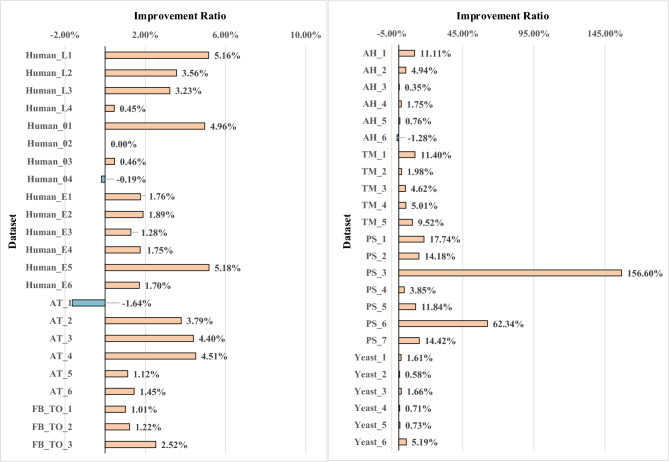
The improvement ratio of 47 datasets

#### Analyze the PrSM Improvement Ratio

To quantitatively analyze the impact of PrSMBooster on the characterization results of each MS dataset, the following formula is applied.

3 $$\begin{aligned} r=\frac{n_{\text{TopPIC+PrSMBooster}}-n_{\text{TopPIC}}}{n_{\text{TopPIC}}} \end{aligned}$$

Here, $n_{\mathrm{TopPIC+PrSMBooster}}$ denotes the quantity of PrSMs reported after PrSMBooster rescoring, while $n_{\textrm{TopPIC}}$ denotes the quantity of initial PrSMs reported by TopPIC. The formula above is used to evaluate the degree of improvement across 47 test datasets and categorizes them based on the improvement ratio, as shown in Fig. 6 and Table 2. The maximum improvement ratio, observed in dataset PS_3, is 156%.

**Table 2:** Results of 47 test datasets

PRIDE ID	Dataset name	Number of TopPIC reported PrSMs	PrSMBooster		
			Number	Improve	Percentage increase
-	FB_TO_1	7137	7226	89	1.25%
	FB_TO_2	7359	7506	147	2.00%
	FB_TO_3	6146	6308	162	2.64%
PXD026178	Human_L1	310	326	16	5.16%
	Human_L2	253	262	9	3.56%
	Human_L3	217	224	7	3.23%
	Human_L4	223	224	1	0.45%
PXD026128	Human_01	141	148	7	4.97%
	Human_02	644	644	0	0.00%
	Human_03	861	865	4	0.47%
	Human_04	538	537	−1	−0.19%
PXD029703	Human_E1	1082	1101	19	1.76%
	Human_E2	1060	1080	20	1.89%
	Human_E3	1173	1188	15	1.28%
	Human_E4	572	582	10	1.75%
	Human_E5	560	589	29	5.18%
	Human_E6	763	776	13	1.70%
PXD042298	AH_1	9	10	1	11.11%
	AH_2	81	85	4	4.94%
	AH_3	283	284	1	0.35%
	AH_4	400	407	7	1.75%
	AH_5	655	660	5	0.76%
	AH_6	234	231	−3	−1.28%
PXD018772	TM_1	912	1016	104	11.40%
	TM_2	758	773	15	1.98%
	TM_3	823	861	38	4.62%
	TM_4	659	692	33	5.01%
	TM_5	609	667	58	9.52%
PXD017382	PS_1	62	73	11	17.74%
	PS_2	141	161	20	14.18%
	PS_3	53	136	83	156.60%
	PS_4	52	54	2	3.85%
	PS_5	76	85	9	11.84%
	PS_6	77	125	48	62.34%
	PS_7	104	119	15	14.42%
PXD034368	AT_1	183	180	−3	−1.64%
	AT_2	211	219	8	3.79%
	AT_3	614	641	27	4.40%
	AT_4	709	741	32	4.51%
	AT_5	978	989	11	1.13%
	AT_6	898	911	13	1.45%
PXD046651	Yeast_1	1681	1708	27	1.61%
	Yeast_2	1718	1728	10	0.58%
	Yeast_3	1690	1718	28	1.66%
	Yeast_4	1694	1706	12	0.71%
	Yeast_5	1639	1651	12	0.73%
	Yeast_6	1676	1763	87	5.19%

**Fig. 7:**
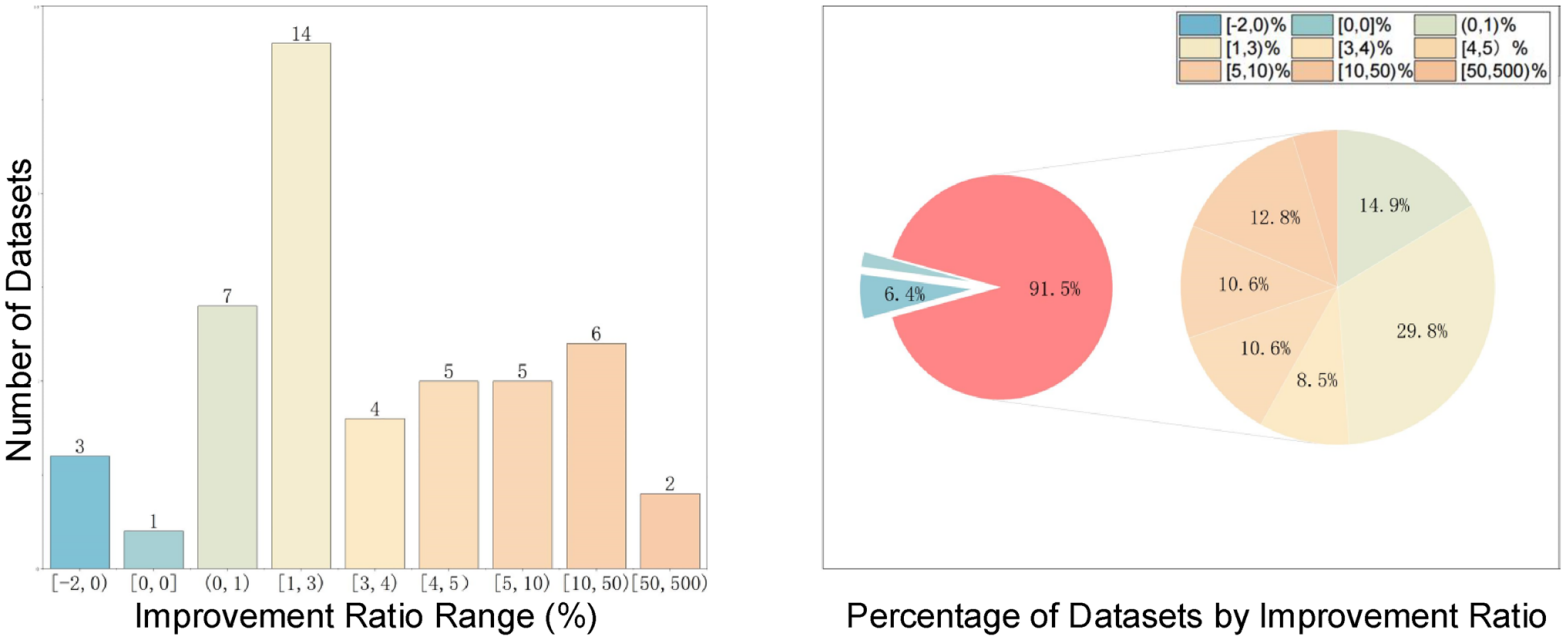
Statistics on the improvement ratio of 47 datasets

Figure 7 shows that among the 47 analyzed datasets, over 91% displayed improvements upon implementing PrSMBooster. Additionally, more than half of these datasets exhibited at least a 3% enhancement compared to the original identification algorithm after rescoring. This suggests the effectiveness of our method in improving the performance of the original characterization algorithm in various scenarios.

**Fig. 8:**
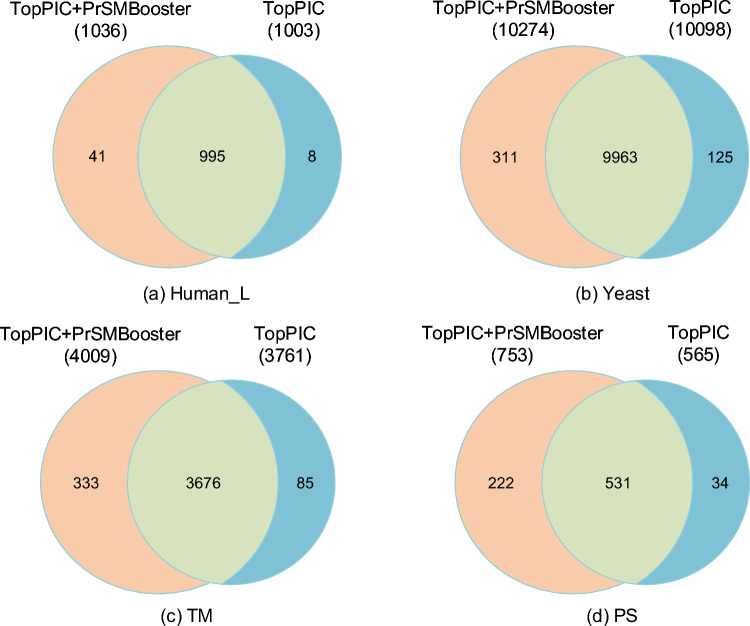
The overlap situation between the results of TopPIC and TopPIC+PrSMBooster

**Fig. 9:**
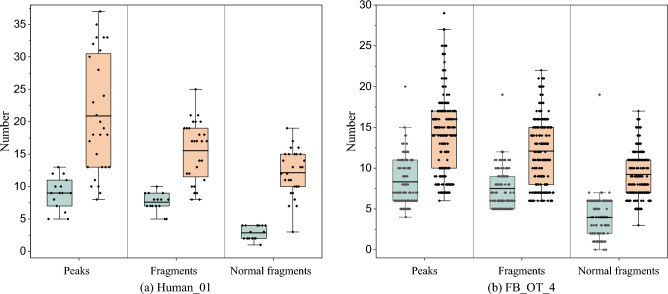
The comparative analysis of unique PrSM features after screening by TopPIC and PrSMBooster on datasets Human_01 and FB_OT_4

### Overlap Analysis

We employed an overlapping analysis method to compare the overlap between the rescoring results of PrSMBooster and the original results of TopPIC, allowing for the quantification of the similarity between our findings and those of previous studies.

#### Coincidence with TopPIC Results

The group of PrSMs under discussion includes PrSMs output by TopPIC and PrSMBooster. It comprises three main categories, PrSMs exclusively output by TopPIC, PrSMs exclusively output by PrSMBooster, and PrSMs output by both algorithms. For brevity, we will refer to them as PrSMBooster-exclusive PrSMs, TopPIC-exclusive PrSMs, and common PrSMs.

Figure 8 depicts the overlap of PrSM results across four datasets (Human_L, Yeast, TM and PS). Firstly, since the number of PrSMs output after rescoring using PrSMBooster exceeded that from the TopPIC algorithm, all datasets exhibited a relatively larger count of PrSMBooster-exclusive PrSMs compared to TopPIC-exclusive PrSMs. Moreover, a considerable percentage of PrSMs were common across the datasets, potentially representing fundamental features. These findings indicate the scientific validity of the PrSMBooster model without distorting the identification outcomes, maintaining the scientific rigor expected from a rescoring model. Notably, the analysis of the datasets highlights the advantage of PrSMBooster in handling large datasets, with a positive correlation observed between dataset size and the proportion of common PrSMs after rescoring.

#### Specificity PrSM Analysis

**Fig. 10:**
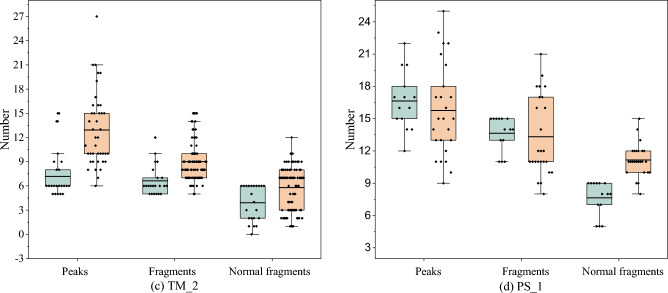
The comparative analysis of unique PrSM features after screening by TopPIC and PrSMBooster on datasets TM_2 and PS_1

The overlap analysis uncovered discrepancies in the PrSM results obtained from TopPIC and TopPIC with PrSMBooster. Figure 9 and Fig. 10 illustrates the statistical characteristics of these non-shared PrSMs across datasets from Human_01, FB_TO_4, TM_2 and PS_1. In top-down MS, fragments are ions detected after the fragmentation of intact protein molecules that correspond to theoretically predicted fragments of the protein sequence. The normal fragments refers to fragments generated from predictable and more common fragmentation pathways, such as cleavages along the peptide backbone. These fragments serve as evidence for the identification of protein sequences or specific proteoforms. PrSMs reported by PrSMBooster exhibited differences in key features, such as matching peaks and fragments, compared to the unique PrSMs identified by TopPIC. In all four datasets, most PrSMs reported by PrSMBooster demonstrated higher values for matching peaks and fragments compared to those reported by TopPIC. This highlights the greater reliability of PrSMs obtained by PrSMBooster compared to those uniquely identified by TopPIC.

**Fig. 11:**
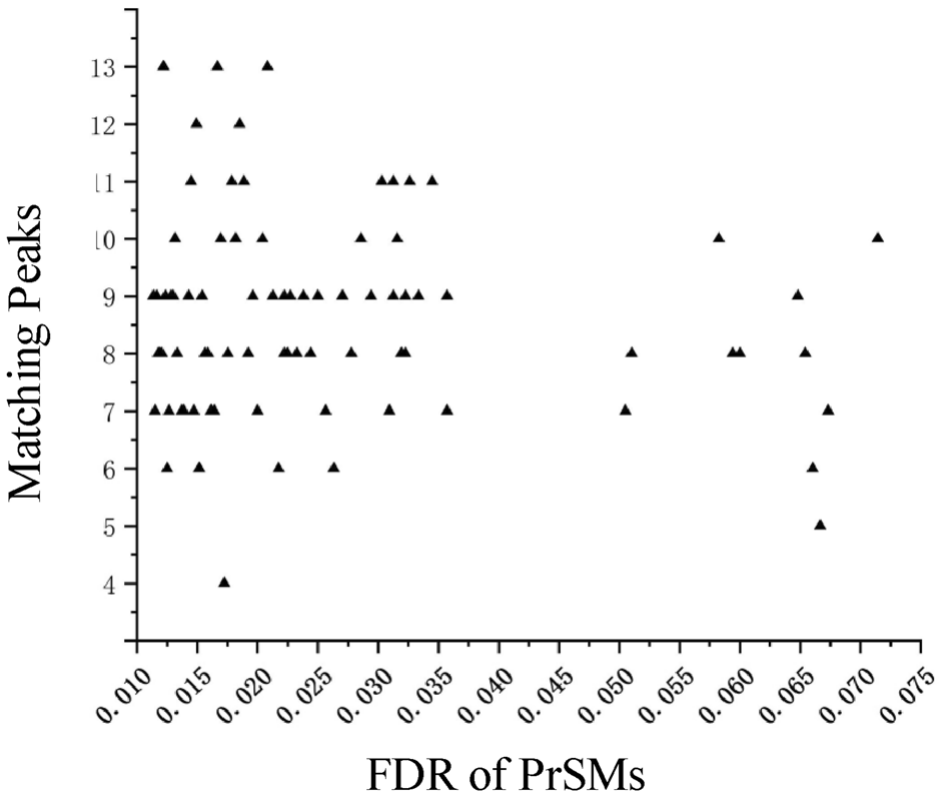
Statistics on the improvement ratio of 47 datasets

Figure 11 illustrates the matching peaks of the exclusive results for pisum sativum (PS_3) following the application of PrSMBooster. TopPIC reported 53 PrSMs, while 136 PrSMs were reported after applying PrSMBooster, encompassing all the PrSMs identified by TopPIC. Analysis of the FDR values of the PrSMBooster results reveals that, after rescoring the original candidate PrSMs, PrSMBooster successfully recaptured several PrSMs with FDR values above 0.05 that had been discarded by the original scoring algorithm. Additionally, it characterized numerous PrSMs with FDR values ranging from 0.01 to 0.045. These PrSMs were excluded in TopPIC due to their FDR values exceeding the threshold of 0.01. Regarding the matching characteristics of the output results, the majority of matching peaks for the output PrSMs fall within the range of 8 to 16. This indicates that the PrSMBooster has a better effect on PrSMs with more than 8 matching peaks.

## Conclusions

This study introduces PrSMBooster, an innovative rescoring model that utilizes traditional machine learning methods to capture latent features. ResNeXt is then used to integrate the basic and newly extracted features for the final PrSM rescoring step. Additionally, experiments on 47 independent datasets shows that PrSMBooster can increase the number of reported PrSMs. Finally, PrSMBooster shows strong potential for improving proteoform characterization accuracy and achieving generalization.

## Data Availability

The zebrafish dataset can be downloaded at https://bit.ly/3pkGB03, and the other datasets can be searched and downloaded by PRIDE ID at https://www.ebi.ac.uk/pride/archive.
